# Dormancy and endosperm presence influence the *ex situ* conservation potential in central European calcareous grassland plants

**DOI:** 10.1093/aobpla/plz035

**Published:** 2019-06-25

**Authors:** Simone Tausch, Martin Leipold, Christoph Reisch, Peter Poschlod

**Affiliations:** Ecology and Conservation Biology, Institute of Plant Sciences, Faculty of Biology and Preclinical Sciences, University of Regensburg, Universitätsstrasse, Regensburg, Germany

**Keywords:** Ageing, grassland, LiCl, *p*_*50*_, physical dormancy, physiological dormancy, seed longevity

## Abstract

The preservation of plant species under *ex situ* conditions in seed banks strongly depends on seed longevity. However, detailed knowledge on this seed ecological aspect is limited and comparative studies from central European habitats are scarce. Therefore, we investigated the seed longevity of 39 calcareous grassland species in order to assess the prospects of *ex situ* storage of seeds originating from a single, strongly threatened habitat. Seed longevity (*p*_*50*_) was determined by artificially ageing the seeds under rapid ageing conditions (45 °C and 60 % eRH (equilibrium relative humidity)), testing for germination and calculating survival curves. We consulted seed and germination traits that are expected to be related to seed longevity. *P*_*50*_ values strongly varied within calcareous grassland species. The *p*_*50*_ values ranged between 3.4 and 282.2 days. We discovered significantly positive effects of physical dormancy and endosperm absence on *p*_*50*_. Physiological dormancy was associated to comparatively short longevity. These relationships remained significant when accounting for phylogenetic effects. Seed mass, seed shape, and seed coat thickness were not associated with longevity. We therefore recommend more frequent viability assessments of stored endospermic, non-physically and physiologically dormant seeds.

## Teaser

Despite increasing efforts towards habitat and species protection in Europe since the 1990s when the Fauna-Flora-Habitat directive was adopted, the proportion of threatened plant species has steadily increased. One strategy to combat this, according to the Global Strategy for Plant Conservation, is to conserve threatened plant species in *ex situ* collections such as seed gene banks. However, the longevity of seeds of wild species under genebanking conditions is not well known. Our study on species from one of the most species-rich and endangered habitats in Europe has shown that there are strong differences in longevity. Concerning seed characters, physical dormancy and endosperm absence were associated with high longevity, whereas physiological dormancy was associated with comparatively short longevity.

## Introduction

The awareness of the importance of seed banks as a tool for *ex situ* conservation of rare and endangered plant species is increasing (Hay and Probert 2013). The subsequent use of seed banks for conservation and restoration management is becoming apparent, regionally (Tausch *et al.* 2015) as well as globally (Godefroid *et al.* 2011; Merritt and Dixon 2011).

Besides the initial viability of a seed lot, knowledge about seed lifespan in storage is essential, as viability decline may not only result in a reduced number of seedlings but also in a loss of genetic diversity. In *ex situ* storage facilities, seeds are preserved under conditions that can extend seed persistence considerably, up to hundreds of years (Walters *et al.* 2005a; Van Treuren *et al.* 2012). More specifically, freezing seeds with low water content (Smith *et al.* 2003) reduces metabolic activity, delays degenerative processes and therefore slows down seed ageing (Walters 1998; Kranner *et al.* 2011). This is valid for orthodox seeds, which are prevalent in the central European flora (Hay and Probert 2013), while recalcitrant seeds do not tolerate drying. Desiccation tolerant seeds possess intrinsic mechanisms to preserve cellular components as water is removed, for example non-reducing sugars, oligosaccharides and Late Embryogenesis Abundant proteins (Bewley *et al.* 2013). However, even orthodox seeds, when stored under optimal conditions, cannot survive indefinitely (Walters *et al.* 2005b). Similar to differences between species in terms of seed bank persistence for different lengths of time when buried in the soil (Kiefer and Poschlod 1996; Bekker *et al.* 1998; Saatkamp *et al.* 2009), there are species-specific differences in storage longevity when seeds are banked (Pritchard and Dickie 2003; Walters *et al.* 2005b; Long *et al.* 2008; Probert *et al.* 2009; Mondoni *et al.* 2011; Merritt *et al.* 2014). Therefore, prioritizing species according to biogeographic criteria or Red Lists is not only important for the selection of target species for collection (Godefroid *et al.* 2011; Griffiths *et al.* 2015) but also for identifying species for regeneration and recollection in certain time intervals (Hay and Probert 2013).

Information about seed bank longevity can be gathered by monitoring and detecting viability decrease of stored seeds over decades (Crawford *et al.* 2007; Probert *et al.* 2009; Godefroid *et al.* 2010) or, more quickly, by using artificial ageing methods (Newton *et al.* 2009). Another method would be to derive predictions based on the viability equations (Ellis and Roberts 1980), but the parameters of these equations have only been determined for a small number of mainly crop species (Hay and Probert 2013), which makes this approach less feasible for wild species. The artificial ageing method induces accelerated seed death by the use of warm and moist conditions, which are literally the opposite of the life extending conditions utilized in *ex situ* facilities. Germinability is measured in regular intervals and the *p*_*50*_ value (time until viability has reached 50 % viability) is determined to enable comparability of seed longevity data (Long *et al.* 2008; Probert *et al.* 2009). Probert *et al.* (2009) showed a highly significant correlation between viability decline of seeds after 20 years in seed bank storage and the mean *p*_*50*_ in artificial ageing.

Therefore, the accelerated ageing method is applied to gain a better understanding of the underlying physiological and biochemical mechanisms of deterioration and repair in plant cells during ageing, which are complex and still not fully understood (Nagel *et al.* 2014). Higher temperature, humidity and oxygen concentration increase the amount of free radicals and reactive oxygen species (ROS), which accumulate during seed ageing (Bailly 2004). These free radicals cause damage to macromolecules such as nucleic acids, lipids, enzymatic and structure proteins, especially in combination with a reduced antioxidant enzyme activity due to ageing (Walters 1998; Bernal-Lugo *et al.* 2000; Bailly 2004; Kranner *et al.* 2011; Nagel *et al.* 2014). Such detailed cellular examinations of viability loss are mainly performed by agricultural seed banks, on one or different genotypes of one (model) species (Walters 1998; Bailly 2004; Kranner *et al.* 2011; Nagel *et al.* 2014). Recently published large comparative longevity studies on wild plant species focus on the influence of the climate of the provenance and seed or plant traits on seed longevity (Long *et al.* 2008; Probert *et al.* 2009; Mondoni *et al.* 2011; Merritt *et al.* 2014). These characteristics may be used to predict seed longevity and assess the prospects of storing seeds in seed banks. It was found that seeds sourced from plants of warmer and drier environments were more long-lived in dry storage (Walters *et al.* 2005b; Probert *et al.* 2009) and rapid ageing assessments (Long *et al.* 2008; Probert *et al.* 2009; Mondoni *et al.* 2011) than those from cooler and wetter climates. For example, seeds collected from alpine populations (with cool wet conditions) were short-lived in comparison with seeds from (related taxa of) lowland populations (Mondoni *et al.* 2011). Merritt *et al.* (2014) confirmed a weak correlation of temperature and *p*_*50*_ for Australian species, but they also found a contradictory result in the form of a negative correlation of annual precipitation and *p*_*50*_. Since the correlations of Probert *et al.* (2009) and Mondoni *et al.* (2011) were relatively weak, rainfall appears to be an unreliable predictor so far (Merritt *et al.* 2014). Regarding the influence of seed traits on seed persistence, seed size and shape as well as dormancy and seed coat thickness have been found to be promising predictors for soil seed bank persistence (Thompson *et al.* 1993; Bekker *et al.* 1998; Hodkinson *et al.* 1998; Funes *et al.* 1999; Peco *et al.* 2003; Thompson *et al.* 2003; Moles and Westoby 2006; Gardarin *et al.* 2010; Schwienbacher *et al.* 2010; Saatkamp *et al.* 2011, 2014; Zhao *et al.* 2011). However, longevity in *ex situ* facilities was not significantly correlated with seed size (Probert *et al.* 2009), or only a slightly positive correlation was found (Merritt *et al.* 2014). Moreover, *Arabidopsis thaliana* showed a negative correlation of dormancy and longevity (Nguyen *et al.* 2012). Endosperm presence or embryo-endosperm ratio were identified as indicators of *ex situ* seed longevity (Walters *et al.* 2005b; Probert *et al.* 2009; Mondoni *et al.* 2011; Merritt *et al.* 2014) and phylogeny also exerted significant influence (Walters *et al.* 2005b; Probert *et al.* 2009; Merritt *et al.* 2014).

The lack of influence of seed morphological traits such as seed size on *ex situ* storage may be explained by the huge geographic range of the investigated species which might mask any habitat-specific effect and alter the significance of these seed traits on longevity (Long *et al.* 2015). Other traits like seed coat thickness and seed shape have not been investigated yet, although they have been shown to be correlated with soil seed bank persistence. To control for climatic effects, a study of seed persistence in a single habitat might reveal the main drivers for *ex situ* seed longevity. To our knowledge, comparative studies on the longevity of seeds in a single habitat, as performed by Tuckett *et al.* (2010) for temporal wet grasslands, are quite rare.

In the present study, we therefore focus on calcareous dry grasslands to explore the ageing rate of seeds of 39 species from one habitat. The habitat was selected because it is the most species-rich in terms of vascular plants and one of the most threatened habitats in Central Europe (Korneck *et al.* 1998; Finck *et al.* 2017). We aimed to explore the influence of seed traits (mass, shape, seed coat thickness, endosperm presence and dormancy) on seed longevity. As recent studies showed no correlation with oil content and carbohydrate composition (Pritchard and Dickie 2003; Walters *et al.* 2005b; Probert *et al.* 2009) and the availability of suitable data is sparse for wild plant species, we did not consider these potential correlates in our analyses. Furthermore, we considered phylogenetic influences on the investigated data to account for relatedness of species.

Considering this background, our study focuses on the following question: Which seed traits influence seed ageing rates of calcareous grassland species and do significant effects remain when statistically testing and accounting for phylogenetic relationships?

## Materials and Methods

### Seeds of calcareous grassland species of Central Europe

Seeds of 39 calcareous grassland species were collected in 2012 in the area of the Jurassic Mountains of the Franconian Alb (Bavaria, southern Germany). The climate can be characterized as a transition climate, with intermediate conditions between mild oceanic climate of western Germany and subcontinental climate in the east (Herbst *et al.* 2014). Mean annual precipitation is 648 mm with summer and winter rains, including heavy snowfalls. Annual mean temperature of 8.4 °C can be described as mild but events like freezing may take place in winter and significantly reduce temperature (Herbst *et al.* 2014).

Species were selected to represent both, the habitat and a broad variation in plant families represented within the flora of Germany. Seeds were freshly collected, cleaned and then stored for 3 months at 4 °C and 40 % humidity before testing. Seed filling and potential viability were assessed via X-ray prior to the ageing experiments. Viability tests applying tetrazolium have shown that the filling rate was equivalent to a nearly 100 % or 100 % viability rate (data not published). Table 1 provides an overview of the 39 species from 18 plant families and 13 orders, and their origin. Additionally we used seeds of *Ranunculus sceleratus* as a marker species for short-lived seeds (Newton *et al.* 2009), with a known *p*_*50*_ (Probert *et al.* 2009).

**Table 1.** Calcareous grassland species used for controlled ageing. Plant families, orders and endosperm presence/absence (N = little or non-endospermic (embryo types FA1–FA4, P); E = abundant endosperm (MA, LA, B1–B4), following Finch-Savage and Leubner-Metzger (2006)) are given. Seed longevity is expressed as *p*_*50*_ (the time to 50 % viability loss) for seeds aged at 45 °C and 60 % RH. Seed longevity for each species is ranked as 1–39, with 1 being the longest-lived species. Pre-treatment refers to the treatment used to break dormancy. SCAR = scarification (after ageing and before germination/viability testing), STRAT = stratification for 6 weeks at 4 °C. Dormancy type—ND = no dormancy, PD = physiological dormancy, PY = physical dormancy. Germination temperature (Germ. Temp.) refers to the constant or daily alternating (14 h day/10 h night) temperature regime and parallel light/darkness fluctuations used for germination testing.

Species	Family (-aceae)	Order (-ales)	Endosperm	Pre-treatment	Dormancy type	Germ. Temp. (°C)	*p*_*50*_ ± SE (days)	Rank
*Achillea millefolium*	Aster-	Aster-	N	–	ND	22/22	46.7 ± 1.4	14
*Acinos arvensis*	Lami-	Lami-	N	–	ND	22/14	28.6 ± 1	24
*Anthericum ramosum*	Asparag-	Lili-	E	STRAT	PD	22/14	45.6 ± 1.2	15
*Anthyllis vulneraria*	Fab-	Fab-	N	SCAR	PY	22/14	198.2 ± 8.4	2
*Arabis hirsuta*	Brassic-	Brassic-	N	–	ND	22/14	63.4 ± 1.6	9
*Arenaria serpyllifolia*	Caryophyll-	Caryophyll-	N	–	ND	22/14	54.3 ± 1.5	12
*Asperula cynanchica*	Rubi-	Gentian-	E	STRAT	PD	22/14	16.2 ± 0.5	31
*Briza media*	Po-	Po-	E	–	ND	22/14	14.8 ± 0.7	32
*Bromus erectus*	Po-	Po-	E	–	ND	22/14	29.3 ± 1.1	22
*Buphthalmum salicifolium*	Aster-	Aster-	N	–	ND	26/18	82.6 ± 1.9	7
*Campanula rotundifolia*	Campanul-	Aster-	E	–	ND	22/14	10.8 ± 0.5	36
*Carduus nutans*	Aster-	Aster-	N	–	ND	22/14	28.4 ± 0.8	25
*Carex flacca*	Cyper-	Po-	E	STRAT	PD	22/14	13.7 ± 0.9	34
*Centaurea stoebe*	Aster-	Aster-	N	–	ND	22/22	52 ± 1.7	13
*Cerastium arvense*	Caryophyll-	Caryophyll-	N	–	ND	14/6	55.7 ± 1.5	11
*Daucus carota*	Api-	Api-	E	–	ND	22/14	44.2 ± 1.7	17
*Dianthus carthusianorum*	Caryophyll-	Caryophyll-	N	–	ND	22/14	42.3 ± 1.2	19
*Galium verum*	Rubi-	Gentian-	E	STRAT	PD	22/14	16.4 ± 1	30
*Genista tinctoria*	Fab-	Fab-	N	SCAR	PY	22/14	73.6 ± 3.1	8
*Globularia bisnagarica*	Plantagin-	Lami-	N	STRAT	PD	22/14	14.8 ± 1.1	33
*Helianthemum nummularium*	Cist-	Malv-	N	SCAR	PY	22/14	155 ± 4	6
*Hypericum perforatum*	Clusi-	Malpighi-	N	–	ND	22/14	29.9 ± 1	21
*Linum catharticum*	Lin-	Malpighi-	N	GA3	PD	22/14	43.5 ± 1.9	18
*Lotus corniculatus*	Fab-	Fab-	N	SCAR	PY	22/14	197.9 ± 5.8	4
*Medicago lupulina*	Fab-	Fab-	N	SCAR	PY	22/14	198.1 ± 35 933.4	3
*Melica ciliata*	Po-	Po-	E	–	ND	22/14	21.9 ± 341.8	28
*Phleum phleoides*	Po-	Po-	E	–	ND	22/14	25 ± 0.8	27
*Pimpinella saxifraga*	Api-	Api-	E	STRAT	PD	22/14	4.9 ± 0.4	38
*Prunella grandiflora*	Lami-	Lami-	N	–	ND	18/10	57.2 ± 1.6	10
*Pulsatilla vulgaris*	Ranuncul-	Ranuncul-	E	–	ND	26/18	31.3 ± 1.6	20
*Rhinanthus minor*	Scrophulari-	Lami-	E	STRAT	PD	22/14	3.4 ± 0.2	39
*Scabiosa columbaria*	Dipsac-	Dipsac-	N	–	ND	22/14	18.8 ± 0.8	29
*Seseli annuum*	Api-	Api-	E	STRAT	PD	22/14	5.4 ± 0.6	37
*Stachys recta*	Lami-	Lami-	N	GA3	PD	22/14	45.1 ± 1.6	16
*Teucrium chamaedrys*	Lami-	Lami-	N	GA3	PD	22/14	29.2 ± 1.4	23
*Teucrium montanum*	Lami-	Lami-	N	GA3	PD	22/14	25.7 ± 0.5	26
*Thymus pulegioides*	Lami-	Lami-	N	–	ND	22/14	12.4 ± 0.5	35
*Trifolium arvense*	Fab-	Fab-	N	SCAR	PY	43 756	282.2 ± 26	1
*Trifolium montanum*	Fab-	Fab-	N	SCAR	PY	22/14	165.8 ± 5.9	5

### Controlled ageing test

Controlled ageing tests were conducted according to the protocol for comparative seed longevity testing (Newton *et al.* 2009; Probert *et al.* 2009). Firstly, for humidity adjustment, seeds were placed in glass vials in a thermoplastic enclosure box (0.3 × 0.4 × 0.102 m; Ensto, Finland) at 20 °C for 14 days over a non-saturated solution of LiCl (EMSURE® ACS, Reag. Ph Eur, Merck, Germany) of 47 % RH (1 L distilled water and 385 g LiCl). The eRH (equilibrium relative humidity) of a dummy sample was measured using a hygrometer (Hygropalm-AW1–AW-DIO, Rotronic, Germany). Secondly, a controlled ageing environment was arranged by placing the seeds in another box in a drying oven at 45 ± 1 °C over a LiCl solution with 60 % RH (1 L distilled water and 300 g LiCl). A sample of 50 seeds was regularly withdrawn and used for germination experiments.

The eRH of a dummy sample and the solutions were regularly checked using the hygrometer. If necessary, the LiCl solution was adjusted by adding distilled water.

### Germination testing

Prior to germination seeds were X-rayed (Faxitron MX 20, Faxitron Bioptics, LLC, Tucson, AZ, USA) to guarantee that none of the seeds were empty or infested. Two replicates of 25 seeds each were germinated under appropriate conditions (see Table 1) sown on two layers of moist (deionized water) filter paper in Petri dishes and placed in climate chambers (Rumed, type 1301, Rubarth Apperate GmbH, Laatzen, Germany) or in a cooling room (4 °C), when pre-chilling was required. The incubators were run with a photoperiod of 14 h light (cool white fluorescent tubes, ±10 000 lux; ~ ±250 µmol·m^−2^·s^−1^ Photosynthetically Active Photon Flux Density) and 10 h darkness. The particular alternating temperatures are shown in Table 1. Light was provided during the warm temperature phase. Four species required additional treatment with GA_3_ (250 mg·L^−1^; Sigma-Aldrich Company Ltd, Dorset, UK) and 11 species with physically dormant seeds were scarified with a scalpel before germination. Seeds were regularly checked for germination and considered viable when germinated—e.g. a radicle protrusion of ≥2 mm occurred and a development of ‘normal seedlings’ was ascertained (Black *et al.* 2006; Bewley *et al.* 2013). Germination test time was at least 42 days; tests were finished after 14 days without germination. At the end of the germination tests, tetrazolium tests were performed to confirm that the viability of ungerminated seeds.

### Seed traits

Seed mass was determined as thousand seed weight extrapolated on the basis of weights of eight samples of 100 seeds each. Seed dimensions were measured on five replicate seeds per species. Seed shape was used as the variance of seed dimensions, which was calculated according to Bekker *et al.* (1998):

$$\begin{array}{l} V_{S}={}^{\overset{}{\sum }{\left(x_{i}-\bar{x}\right)}^{2}}╱{}_{n}\; \end{array}$$ (1)

where *x*_1_ = length/length, *x*_2_ = height/length and *x*_3_ = width/length, *n* = 3. Seed shape is a dimensionless trait that varies between 0 in perfectly round and 0.2 in disk- or needle-shaped seeds. Endosperm presence/absence was determined by X-ray analysis, dissection and the classification according to Martin (1946), revised and extended by Finch-Savage and Leubner-Metzger (2006). Seeds with peripheral embryo were classified as non-endospermic seeds, as they had a higher embryo to seed ratio than seeds with abundant endosperm (endospermic basal embryo types B1, B3 and B4, phylogenetically more advanced endospermic seeds LA, MA according to Finch-Savage and Leubner-Metzger (2006)). Prior germination tests allowed us to identify whether seeds possessed physical or physiological dormancy (see Table 1). Seed coat thickness was determined as mean seed coat thickness of five seeds using X-ray photographs in an image processing programme. We were not able to measure seed coat thickness of four species (*Dianthus carthusianorum*, *Bromus erectus*, *Melica ciliata* and *Phleum phleoides*), as the seed coat or testa plus pericarp were not visible. These species therefore had to be excluded from some statistical analyses.

### Data analysis

Statistical analyses, unless stated otherwise, were performed using R version 3.1.1 (R Development Core Team).

### Viability curves and assessment of *p50* values

For the calculation of *p*_*50*_ we applied a slightly modified definition of this value. In most papers, *p*_*50*_ is defined as the value when viability has fallen to 50 % of total viability. The precondition of this approach is that seeds should have a high (>85 %) initial viability and germination requirements must be known (Newton *et al.* 2009). However, when initial viability is lower, it is recommended to calculate *p*_*50*_ as 50 % of initial viability (Walters *et al.* 2005b). However, to apply two kinds of definitions which require two different statistical calculations is not useful instead of calculating consequently *p*_*50*_ as 50 % of initial viability. Since the *p*_*50*_ values of 50 % viability at high initial germination percentages do not differ strongly from the *p*_*50*_ values of 50 % of initial viability, we suggest to apply consequently this approach in future studies.

For the statistical calculation of *p*_*50*_, two approaches were adopted. The first was a probit analysis that fits the seed viability equation of Ellis and Roberts (1980):

$$\begin{array}{l} \upsilon =K_{i}-p/\sigma \end{array}$$ (2)

where *υ* is the viability in normal equivalent deviates (NED) at time *p* (days); *K*_*i*_ is the initial viability (NED) and *σ* is the standard deviation of the normal distribution of seed deaths in time. The probit analysis was performed using both the statistics software Genstat 11th edition (Payne *et al.* 2008) and the drc package in R (Ritz and Streibig 2005) especially for drawing the viability curves. Both packages produced the same results.

As a second approach, we fitted curves using the equation (3) of Long *et al.* (2008), which provides the fitted initial germination percentage (100 − *α*), the rate of viability loss in the rapidly declining section of the curve (*β*), the accumulated time in the ageing environment (*t*) and the *p*_*50*_ value (*c*). However, negative logistic (sigmoidal) curves were not suitable for all species.

$$\begin{array}{l} Germination(\;\%\;)=(100-\alpha )/[1+e^{-\beta (t-c)}] \end{array}$$ (3)

At the end, probit analysis has resulted in the best fit for all species. Therefore, these data were used for any further calculation.

### Phylogeny

The phylogenetic tree required for the phylogenetic analysis was constructed using Phylomatic v3 (Webb and Donoghue 2005) based on the megatree R20120829 APG III (2009). Nodes of the phylogeny were then dated according to Wikstrom *et al.* (2001) and attached to the phylogeny using BLADJ, returning a new phylogeny with adjusted branch lengths (Webb *et al.* 2008).

### Transformations and phylogenetic signals of seed traits and *p50*

Due to non-normality (Shapiro–Wilk tests), *p*_*50*_, seed mass (TSW), seed shape (*V*_*S*_) and mean seed coat thickness (MCT) were log_10_-transformed in order to gain normal distributed data. As closely related species tend to share phenotypic similarities, which they inherited from ancestors, direct correlation studies that treat each species as an independent data point may increase the risk of Type I errors and thus lead to incorrect rejection of the null hypothesis (Freckleton *et al.* 2002). Therefore, it is advised to account for dependencies due to relatedness of species by using phylogenetic comparative methods (Garland and Ives 2000; Freckleton *et al.* 2002; Garland *et al.* 2005).

To quantify for phylogenetic signals in our continuous variables, we used two alternative parameters: Pagel’s *λ* (Pagel 1999) and Blomberg’s *K* (Blomberg *et al.* 2003). In addition, for the binary traits endosperm persistence, physical dormancy and physiological dormancy, we used Fritz and Purvis’ *D* (Fritz and Purvis 2010). All three phylogenetic parameters evaluate the signal in a trait against a Brownian motion model of trait evolution. In the Brownian motion model, trait evolution follows a random walk along the branches of the phylogenetic tree, with time being represented by branch lengths and the trait being directly proportional to the branch length/time (Revell *et al.* 2008). For continuous valued traits under a pure Brownian motion evolution, the expected covariance between the trait values of species at the tips of the phylogeny is proportional to the lengths of shared branch lengths (off-diagonals, Ives and Garland 2010).

For *λ* and *K*, a value of 0 reveals that the variation of a trait is modelled as a function of independent evolution (star phylogeny, no phylogenetic signal), while values of 1 show that the variation of a trait is as expected under the Brownian model (strong phylogenetic signal). *K* can exceed 1, which indicates a greater degree of trait similarity among related taxa than expected under Brownian motion. *K* and *λ* were calculated using the phylosig function in the phytools package (Revell 2012) and *λ* was additionally estimated using the pgls function in the caper package (Orme *et al.* 2012) with a maximum likelihood approach. For *λ* both packages produced the same results.

*D* statistic was carried out with the phylo.d function in caper. Here, 0 indicates that a trait evolves on a tree following the Brownian model and 1 indicates that the trait evolves following a star phylogeny. A negative *D* indicates a trait that is more conserved than predicted by the Brownian model. Additionally we conducted a simulation (1000 permutations) to test whether an estimated *D* was significantly different from the predictions of a random or a Brownian evolution.

### Conventional statistical analysis of seed trait correlates of *p50*

For our first set of analyses, we used non-phylogenetic methods that assume species to be related by a star phylogeny, e.g. that there is no phylogenetic structure and all species being equally related (Felsenstein 1985; Perry and Garland 2002; Blomberg *et al.* 2003). Relationships between *p*_*50*_ and the seed traits seed mass, seed shape, mean coat thickness, endosperm presence/absence, physical dormancy presence/absence and physiological dormancy presence/absence were examined through generalized least squares regression analyses, using maximum likelihood estimation, using single traits and different combinations as predictors. Models were compared using the small unbiased Akaike Information Criterion (AIC_c_) and the Akaike weight (*w*_*i*_). Finally, we computed the model-averaged predictions as weighted means, where *w*_*i*_ served as model probabilities (Burnham and Anderson 2002). We also compared *p*_*50*_ values of non-dormant, physically and physiologically dormant seeds, using two one-way ANOVAs and subsequent Tukey’s HSD *post hoc* analyses.

### Phylogenetic analysis of seed trait correlates of *p50*

Where we found phylogenetic signals, we used a phylogenetic generalized least squares model (PGLS; Grafen 1989; Pagel 1999; Freckleton *et al.* 2002) to correct for phylogenetic non-independence. Phylogenetic generalized least squares model is capable of evaluating multiple predictor variables and incorporating polytomies (Pagel 1992) and is regarded as the most general robust way of correcting for non-independence in data (Freckleton *et al.* 2002). Here, estimated *λ* was used not only for measuring strength of phylogenetic signal, but also for optimizing internal branch length transformation using maximum likelihood. Model comparison was conducted likewise for the non-phylogenetic models.

## Results

### Seed viability decline of calcareous grassland species in controlled ageing

**Figure 1.**
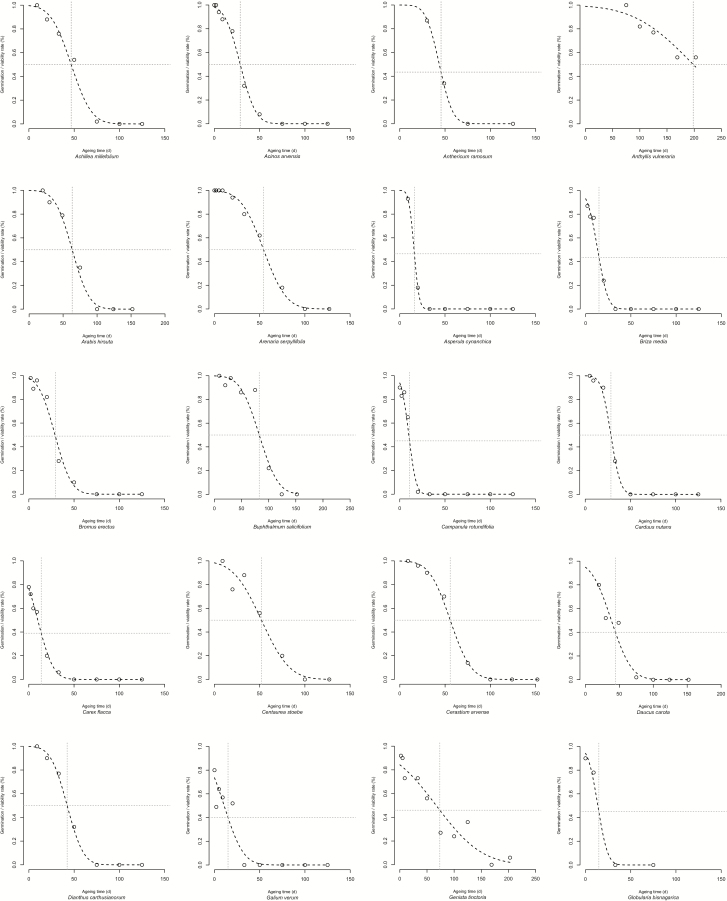

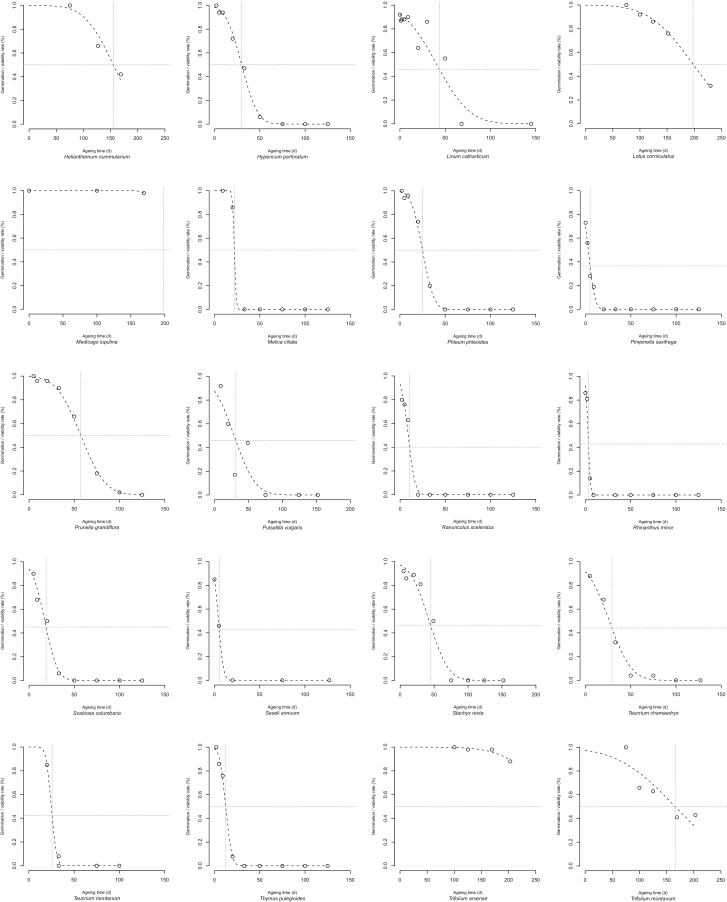
Seed survival curves of calcareous grassland species in controlled ageing at 60 % RH and 45 °C. Curves were fitted by probit analysis (dashed lines). Reference species: *Ranunculus sceleratus*.

Seed viability loss curves over time of the examined species showed different curve progressions, as shown in Fig. 1. The estimate of *p*_*50*_ of calcareous grassland species ranged from 3.4 ± 0.2 days for *Rhinanthus minor* to 282.2 ± 26 for *Trifolium arvense*. In general, species with physically dormant seeds had higher longevity than other species (Table 1). Three Fabaceae species, *Anthyllis vulneraria*, *Medicago lupulina* and *T. arvense* had not yet reached *p*_*50*_ when the experiment ended after 210 days (Fig. 1). In these cases extrapolated *p*_*50*_ values resulting from curve fitting served as approximations. Some species displayed a near-perfect fit to the sigmoidal model like *Arenaria serpyllifolia* (*p*_*50*_ of 54.3 ± 1.5 days) and *D. carthusianorum* (42.4 ± 1.2), other species such as *Seseli annuum* (5.3 ± 0.6) and *Thymus pulegioides* (12.4 ± 0.5) showed very steep viability losses (Fig. 1).

Plant orders can be arranged in order of increasing seed longevity (mean *p*_*50*_) as follows: Gentianales (16.3 ± 0.1, *n* = 2), Apiales (18.2 ± 13, *n* = 3), Dipsacales (18.8 ± 0, *n* = 1), Poales (20.9 ± 3, *n* = 5), Lamiales (27 ± 6.2, *n* = 8), Ranunculales (31.3 ± 0, *n* = 1), Malpighiales (36.7 ± 6.8, *n* = 2), Asterales (44.1 ± 12.1, *n* = 5), Liliales (45.6 ± 0, *n* = 1), Caryophyllales (50.8 ± 4.3, *n* = 3), Brassicales (63.4 ± 0, *n* = 1), Malvales (155 ± 0, *n* = 1) to Fabales (186 ± 27.5, *n* = 6). Within the Apiaceae (*n* = 3) a large variation in *p*_*50*_ was observed, with *Daucus carota* being relatively long-lived (44.2 ± 1.7), *Pimpinella saxifraga* and *S. annuum* being very short-lived (4.9 ± 0.4 and 5.4 ± 0.6). In contrast there was a low variation within the Caryophyllales (*n* = 3) with relatively consistent values for *A. serpyllifolia* (54.3 ± 1.5), *Cerastium arvense* (55.7 ± 1.5) and *D. carthusianorum* (42.3 ± 1.2). The reference species *R. sceleratus* possessed a *p*_*50*_ of 10.5 ± 0.5 days.

### Phylogenetic signals

The survey of phylogenetic signals revealed phylogenetic influences in both, dependent and independent variables (Table 2). Two binary traits showed high significant phylogenetic signals: endosperm presence (*D* = −0.946) and physical dormancy (*D* = −2.185). Except for seed coat thickness, all continuous seed traits showed relatively strong phylogenetic signals although the outputs were significantly different from a Brownian motion model and not significantly different from a star phylogeny considering *λ*.

**Table 2.** Tests of the phylogenetic signals in seed traits and seed longevity for 35 species. Values of *λ* and *K* close to 1 indicate a strong phylogenetic signal; values close to 0 indicate absence of phylogenetic signal in the trait. Values of *D* close to 0 indicate a strong phylogenetic signal, negative values show a stronger conservation than predicted by the Brownian model.

		Pagel’s *λ*			Blomberg’s *K*		Fritz and Purvis’ *D*		
		Difference from					p		
Trait	*n*	*λ*	0	1	*K*	*P*	*D*	star	BM
Seed shape	35	0.633	0.143	0.002	0.497	0.072			
Seed mass	35	0.837	0.152	0.051	0.568	0.029			
Seed coat thickness	35	0.000	1.000	0.001	0.447	0.147			
*p*_*50*_	35	0.744	<0.001	0.015	0.780	0.001			
Endosperm presence	35						−0.917	0.000	0.924
Non-dormancy	35						0.240	0.021	0.327
Physical dormancy	35						−2.060	0.000	0.996
Physiological dormancy	35						0.117	0.015	0.441

### Influence of seed traits on *p50* of calcareous grassland species

**Figure 2.**
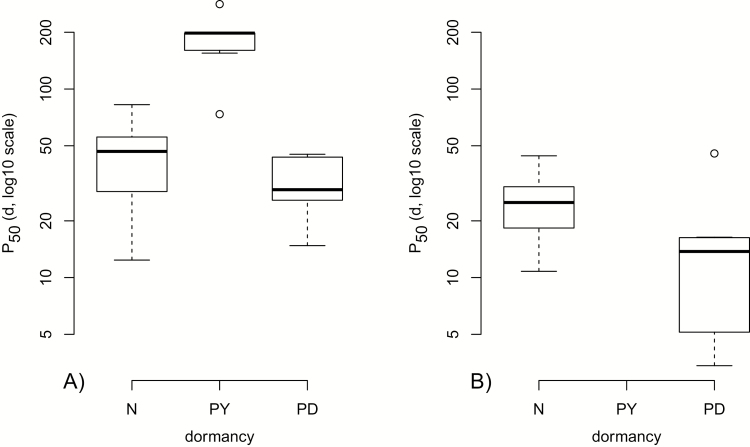
Box plots of *p*_*50*_ values of endospermic (A) and non-endospermic species (B) including hard-coated seeds (non-dormant, N, *n* = 13; physically dormant, PY, *n* = 5; physiologically dormant, PD, *n* = 7) (B) excluding hard-coated seeds (non-dormant, N, *n* = 7; physiologically dormant, PD, *n* = 7). Box plots show the 25–75th percentiles, whiskers span the 10 and 90th percentiles and circles span the 5 and 95th percentiles.

The comparison of all non-phylogenetic models to analyse the influence of seed traits on *p*_*50*_ suggests that the model including all seed traits gave the best fit (AIC_c_ = 20.35, *w*_*i*_ = 0.95, Table 3). The average model of the non-phylogenetic analysis showed significant effects of endosperm presence/absence, physiological dormancy and physical dormancy on *p*_*50*_ (see Table 4). With a mean *p*_*50*_ of 20.20 ± 3.62 days (*n* = 14) and 80.06 ± 14.69 days (*n* = 25; one-way ANOVA, *F* = 20.63, *P* < 0.001) endospermic seeds were significantly shorter-lived than non-endospermic seeds. Even after removing physically dormant seeds the *p*_*50*_ values remained significantly different (one-way ANOVA, *F* = 12.85, *P* = 0.001) with 20.20 ± 3.62 days (*n* = 14) in endospermic and 40.60 ± 4.38 days (*n* = 18) in non-endospermic seeds. Within the non-endospermic seeds, dormancy had a highly significant influence on *p*_*50*_ (one-way ANOVA, *F* = 25.77, *P* < 0.001, Fig. 2): physically dormant seeds were significantly longer-lived than non-dormant or physiologically dormant seeds (post-ANOVA Tukey’s HSD, *P* < 0.001 for both comparisons), but there was no significant difference between physiologically dormant and non-dormant seeds (post-ANOVA Tukey’s HSD, *P* = 0.508). Within endospermic seeds, non-dormant seeds were nearly significantly longer-lived than physiologically dormant seeds (one-way ANOVA, *F* = 4.093, *P* < 0.066).

**Table 3.** Non-phylogenetic and phylogenetic candidate models to explain variation for the *p*_*50*_ values of 35 calcareous grassland species by seed traits compared to the null model (i.e. no explanatory variables). In the phylogenetic analysis, *λ* was used for optimizing internal branch length transformation using maximum likelihood. The number of estimated parameters in each model (*K*), AIC_c_ values for each model, differences in AIC_c_ between each model and the best-fit model (Δ*i*) and the Akaike weight (*w*_*i*_) are displayed. Seed shape (*V*_*S*_), seed mass (TSW) and mean coat thickness (MCT) were log10-transformed. Endo = endosperm presence/absence, PY = physical dormancy, PD = physiological dormancy.

Candidate model	*λ*	*K*	logLik	AIC_c_	Δ*i*	*w*_*i*_
Non-phylogenetic analysis						
*V*_*S*_. TSW. MCT. endo. PD. PY		8	0.60	20.35	0.00	0.95
PY		3	−9.97	26.72	6.38	0.04
MCT. PY		4	−9.93	29.20	8.86	0.01
Endo		3	−14.63	36.04	15.69	0.00
PD		3	−16.76	40.30	19.95	0.00
*V*_*S*_		3	−20.51	47.79	27.44	0.00
*V*_*S*_. TSW		4	−20.38	50.09	29.75	0.00
Null model		2	−22.88	50.13	29.78	0.00
MCT		3	−22.75	52.28	31.93	0.00
TSW		3	−22.77	52.31	31.96	0.00
						
Phylogenetic analysis						
*V*_*S*_. TSW. MCT. endo. PD. PY	0.000	7	0.60	16.96	0.00	0.89
Endo	0.581	2	−8.84	22.05	5.10	0.07
PY	0.000	2	−9.97	24.32	7.37	0.02
MCT. PY	0.000	3	−9.93	26.64	9.69	0.01
PD	0.764	2	−11.54	27.46	10.51	0.00
*V*_*S*_	0.699	2	−13.89	32.15	15.20	0.00
Null model	0.744	1	−15.17	32.46	15.50	0.00
MCT	0.737	2	−14.77	33.92	16.96	0.00
*V*_*S*_. TSW	0.705	3	−13.77	34.32	17.36	0.00
TSW	0.745	2	−15.09	34.56	17.60	0.00

**Table 4.** Regression results for the non-phylogenetic and phylogenetic general least squares models for *p*_*50*_ of 35 calcareous grassland species computed by model averaging. The estimates, standard errors of the estimates, *z-*values and estimated *P-*values (Pr(>|z|)) are given. Seed shape (*V*_*S*_), seed mass (TSW) and mean coat thickness (MCT) were log10-transformed. In the phylogenetic analysis, *λ* was used for optimizing internal branch length transformation using maximum likelihood.

Model-averaged coefficients	Estimate	SE	*z-*value	Pr(>|*z*|)
Non-phylogenetic analysis				
(Intercept)	1.04540	0.52434	1.910	0.0562
*V*_*S*_	−0.38105	0.25553	1.427	0.1536
TSW	0.04752	0.13840	0.329	0.7425
MCT	0.10468	0.27017	0.371	0.7108
Non-endospermic	0.31866	0.11914	2.559	0.0105*
Physical dormancy	0.47438	0.19567	2.338	0.0194*
Physiological dormancy	−0.32750	0.12991	2.412	0.0159*
				
Phylogenetic analysis				
(Intercept)	1.05727	0.51225	2.060	0.0394*
*V*_*S*_	−0.38108	0.25554	1.491	0.1359
TSW	0.04752	0.13839	0.343	0.7313
MCT	0.10487	0.27029	0.388	0.6980
Non-endospermic	0.33644	0.13687	2.458	0.0140*
Physical dormancy	0.46725	0.18973	2.463	0.0138*
Physiological dormancy	−0.32762	0.12989	2.522	0.0117*

*Significant effects of the respective trait on p50 (see text).

Seed shape ranged from 0.019 in *Lotus corniculatus* to 0.179 in *B. erectus*. Seed mass varied between 0.053 mg in *Campanula rotundifolia* and 5.132 mg in *B. erectus* and seed coat thickness between 0.021 mm in *T. pulegioides* and 0.173 mm in *Teucrium chamaedrys*. *P*_*50*_ was influenced neither by seed mass or shape nor by seed coat thickness (Table 4).

AIC_c_ comparison of all phylogenetic models ranked the model including all seed traits highest (AIC_c_ = 16.96, *w*_*i*_ = 0.89), but it was not significantly different from the model only including endosperm presence/absence (see Table 3). The phylogenetic model did not markedly differ from the non-phylogenetic model (see Table 4).

## Discussion

Under conditions of artificial ageing, seed longevity (*p*_*50*_) of calcareous grassland species varied from 3.4 to 92.77 days (290.2 days including hard-coated seeds). Our results were consistent with the longevity *p*_*50*_ values of Northern Italian species from different habitats that ranged from 4.7 to 95.4 days (Mondoni *et al.* 2011). However, in two large studies with Australian species (Merritt *et al.* 2014) or with a global scope (Probert *et al.* 2009), species’ seed longevities reached *p*_*50*_ values of 588.6 and 771 days, respectively. Long *et al.* (2008) also determined higher longevities for Australian than for Western European species. Obviously, warmer and drier climates are bearing larger proportions of long-lived seeds (Walters *et al.* 2005b; Long *et al.* 2008; Probert *et al.* 2009; Mondoni *et al.* 2011). Likewise, on a smaller geographic scale, climatic characteristics (precipitation and temperature) influence seed longevity, e.g. alpine populations possessed more short-lived seeds than lowland populations (Mondoni *et al.* 2011). Based on a logarithmic scale to categorize species according to their relative seed longevity, the majority of 30 species could be classified as having medium-lived seeds in artificial ageing, three as short-lived and six as long-lived (Mondoni *et al.* (2011): ‘very short’ (*p*_*50*_ ≤ 1), ‘short’ (1 < *p*_*50*_ ≤ 10), ‘medium’ (10 < *p*_*50*_ ≤ 100), ‘long’ (100 < *p*_*50*_ ≤ 1000) and ‘very long’ (*p*_*50*_ > 1000).

Regarding plant families or orders, our *p*_*50*_ values confirmed the results of other studies for Apiaceae (Walters *et al.* 2005b; Merritt *et al.* 2014), Campanulaceae and Poales (Probert *et al.* 2009; Mondoni *et al.* 2011) possessing relatively short-lived and Caryophyllaceae or Fabales (Probert *et al.* 2009; Merritt *et al.* 2014) possessing long-lived seeds. Nevertheless, most other families produced species with wide-ranging longevities. These studies imply a phylogenetic basis of seed persistence and capture also the well-known variations in seed persistence according to the pre-harvest environment of the provenance (Ooi *et al.* 2009; Probert *et al.* 2009; Kochanek *et al.* 2011; Mondoni *et al.* 2011; Walck *et al.* 2011). This can lead to the phenomenon that seed provenances of the same species differ in seed longevity as a result of environmental selection (Kochanek *et al.* 2011; Mondoni *et al.* 2011).

We herein investigated the influence of seed mass, seed shape, seed coat thickness, seed dormancy, endosperm presence/absence, also taking into account phylogenetic constraints on *p*_*50*_. *P*_*50*_ itself showed a strong phylogenetic signal, indicating that seed longevity is determined by traits that possess a high phylogenetic signal themselves. This applied to seed endosperm presence and physical dormancy, seed mass and seed shape, which all showed dependencies due to relatedness of species. While endosperm is more abundant in basal plant groups, Finch-Savage and Leubner-Metzger (2006) showed that gain and loss of physiological dormancy occurred several times and at several levels of seed evolution. The strong influences of endosperm presence, (physiological dormancy) and physical dormancy on *p*_*50*_ were still existent when we corrected for phylogenetic non-independence. This again indicates that although these traits exhibit phylogenetic signals, they can also be highly variable in shared clades. It becomes evident, as abundant endosperm is existent as well in basal endospermic plant families such as Poaceae and Ranunculaceae as in more advanced endospermic plant families like Apiaceae and Scrophulariacae. Probert *et al.* (2009) and Merritt *et al.* (2014) also focused on the role of endosperm showing that non-endospermic seeds persist longer. Seeds with small embryos and endosperm are basal among angiosperms (Forbis *et al.* 2002; Finch-Savage and Leubner-Metzger 2006) which led Probert *et al.* (2009) to the assumption that the moist environment of the early angiosperms accounts for the poor longevity of endospermic seeds as seeds did not have to rely on long-term survival in a dry state. As a consequence of increasing seasonality and aridity or colonization of hotter and drier environments, competitive seeds with larger embryos and an orthodox (desiccation tolerant) behaviour might have evolved (Kranner *et al.* 2010). Surprisingly, this strong effect has not been reported for the 69 species of alpine and lowland species in the study of Mondoni *et al.* (2011).

Unlike to soil seed bank persistence (Gardarin *et al.* 2010), seed coat thickness did not influence *p*_*50*_, whereas physically dormant seeds stood out due to their high *p*_*50*_ values. Merritt *et al.* (2014) even showed that water impermeability of the seed coat itself did not contribute to high longevity of physically dormant seeds, as in their study the investigated seeds were scarified prior to artificial ageing. These findings support the assumption of the evolution of non-endospermic seeds together with hard water impermeable seed coats and a high intrinsic longevity. Whereas physical dormancy proofed to be effective in extending seeds’ longevity, physiologically dormant seeds possessed reduced longevity, which was significant for endospermic seeds. This pattern differs from patterns observed in studies of natural seed bank persistence, which found that reduced germinability due to dormancy boosts persistence (Saatkamp *et al.* 2011). However, our results are in agreement with a recent QTL (quantitative trait loci) study on *A. thaliana*, which demonstrated that seed dormancy and seed longevity QTLs were co-located and negatively correlated, using both, artificially and naturally aged seeds (Nguyen *et al.* 2012). In accelerated ageing, seed water contents of 75–100 % RH enable enzyme activity and metabolism (Bewley *et al.* 2013). But as antioxidant and regeneration mechanisms are only sufficiently active in fully imbibed seeds, ROS accumulate uncontrolledly as by-products of aerobic metabolism (Bailly 2004; Bailly *et al.* 2008). When imbibed for germination, excessive ROS amounts lead to oxidative damages and finally seed death in aged seeds (Bailly *et al.* 2008; Bewley *et al.* 2013). In seeds that have not been exposed to ageing, a balanced increasing ROS level is correlated with germination and dormancy release, which is ascribed to an interaction with dormancy-releasing hormones (Bailly *et al.* 2008). Moreover, simultaneously, cell repair is activated (Bewley *et al.* 2013) and germinating (non-dormant) seeds produce protective antioxidants that counteract this excessive ROS activity (Haslekås *et al.* 2003). Dormant seeds do not produce these germination-specific antioxidants. In dormant aged seeds, where ROS is already elevated, this may be fatal even before germination is initiated. These findings may not obligatorily affect all dormant seeds stored in seed banks as it has repeatedly been shown that some seeds may overcome dormancy by cold storage temperatures (Perez-Garcia *et al.* 2007; Mira *et al.* 2011; Van Treuren *et al.* 2012).

Considering seed size measures, seed mass and seed shape, we found no influence on seed longevity. While in context of *ex situ* longevity seed shape has not been studied so far, the lack of influence of seed mass was consistent with findings of Walters *et al.* (2005b) and Probert *et al.* (2009). However, Merritt *et al.* (2014) found a slight but significant correlation of seed mass and *p*_*50*_ and ascribed this finding to the fact that their analysis was based specifically on a larger sample of large-seeded species than other studies. In soil seed banks seed shape as well as seed mass have been shown to be of significance (e.g. Bekker *et al.* 1998). Seed mass may play a role in the soil as predation is more likely and additionally, the trade-off between seed size and seed number may reduce the detection of bigger seeds and therefore causes misinterpretation (Saatkamp *et al.* 2009). These factors are irrelevant in artificial ageing conditions, although one might assume that oxidative damage may be more pronounced in large, flattened seeds (Kranner *et al.* 2010) due to stressful conditions of high temperature and humidity and may additionally overburden repair mechanisms during imbibition.

## Conclusions

As *p*_*50*_ values differed strongly within one habitat, there is no potential for a general advice to curators of storage facilities for an adequate storage of species of calcareous grasslands. By investigating in a single habitat, calcareous grasslands, we attempted to eliminate the potential influence of climate differences that may have masked the significance of traits in other studies. However, we showed that at least two seed traits can provide guidance: physical dormancy (e.g. Fabaceae) and endosperm absence significantly promote storage persistence. We therefore confirmed previous results of geographically more large-scale studies (Walters *et al.* 2005b; Probert *et al.* 2009; Mondoni *et al.* 2011; Merritt *et al.* 2014), implicating the major influence of intrinsic seed characters exceeding the importance of climate. Viability assessment and recollection of stored seeds possessing one or more of these characteristics can be postponed in favour of species with different features. According to FAO (2013) viability should be checked regularly in 5-year intervals to enable regeneration or recollection of seeds. Seeds that are expected to have rapid deterioration rates should also be considered for cryostorage. Seed bank curators must be also aware of the fact that longevity of different accessions of one species can be variable due to the predispersal environment (Kochanek *et al.* 2011).

## Sources of Funding

The Bavarian Agency for Environmental Protection (LfU Bayern) funded the project (project number P.5301).

## Contributions by the Authors

S.T., C.R., and P.P. designed the study. S.T., and M.L. collected the seeds. S.T. did the investigation and together with M.L. the data analysis. P.P. supervised the study. S.T. was writing the original draft. M.L., C.R., and P.P. contributed by reviewing and editing.

## Conflict of Interest

None declared.
